# Medical News

**Published:** 1852-08

**Authors:** 


### MEDICAL NEWS.

Dr. EDWARD HARTSHORNE has been elected Surgeon to Wills' Hospital for diseases of the eyes and limbs, in place of Dr. Neill resigned.

HOSPITAL OF THE PROTESTANT EPISCOPAL CHURCH IN PHILADELPHIA.—The board of managers have recently appointed the following gentlemen as members of the hospital staff.

*Attending Physicians.*—Francis West, M. D., Jno. B. Biddle, M. D. Francis G. Smith, M. D., John J. Reese, M. D.

*Attending Surgeons.*—Wm. B. Page, M. D., H. H. Smith, M. D., B. Henry, M. D., H. Drayton, M. D.

*Obstetricians.*—Anthony E. Stocker, M. D., Jno. Wiltbank, M. D.

*Assistant Physicians.*—A. Wilcocks, M. D., R. Clements, M. D., F. W. Lewis, M. D., Moreton Stillé, M. D.

*Assistant Surgeons.*—A. Hewson, M. D., W. Hunt, M. D., R. F. Penrose, M. D., J. C. Patterson, M. D.

A lot of ground, with a convenient building, has been presented to the Board by Miss Leamy of this city, and it is their intention to commence operations immediately.

THE SAINT JOSEPH'S HOSPITAL.—At a meeting of the Managers of this Institution, on the 12th inst., Dr. Joseph Leidy was unanimously appointed Pathologist to this institution.

Dr. HORNER, Professor of Anatomy in the University of Pennsylvania, has recently met with what may be called an aortico-œsophageal muscle, which arose from the descending thoracic aorta, and proceeding transversely, was attached to the œsophagus about two and a half inches below the division of the trachea. It was about eight lines wide. He has met in his dissections with two instances of the broncho-œsophageal muscle, of Professor Hyrtl, of Vienna. The aortico-œsophageal belongs to the same category of muscular attachment of the œsophagus to viscera of the thorax.

